# First‐Trimester Bilateral Choanal Atresia as a Marker of a De Novo Pathogenic KMT2D Variant Associated With BCAHH Syndrome

**DOI:** 10.1002/pd.70227

**Published:** 2026-07-26

**Authors:** Patrik Šimják, Jan Král, Dagmar Rašková, Monika Koudová, David Stejskal

**Affiliations:** ^1^ Department of Fetal Medicine GENNET Prague Czech Republic; ^2^ Department of Gynecology Obstetrics and Neonatology First Faculty of Medicine Charles University Prague Czech Republic; ^3^ Department of Genetics GENNET Prague Czech Republic; ^4^ GNTLABS By GENNET Prague Czech Republic

## Abstract

- What is already known about the topic?
  ◦ Prenatal detection of choanal atresia is uncommon and, to our knowledge, has not been reported in the first trimester.
  ◦ Similarly, antenatal identification of the recently described BCAHH syndrome has not been previously reported.
- What does this study add?
  ◦ This study provides the first description of a first‐trimester sonographic appearance suggestive of choanal atresia, enabling the first reported prenatal diagnosis of BCAHH syndrome, a rare and severe condition caused by a pathogenic *KMT2D* gene variant.
  ◦ It demonstrates the feasibility of ultrasound detection of choanal atresia and may aid in the prenatal recognition of this malformation and associated syndromic conditions.

## 1 Fetal Phenotype

**FIGURE 1:**
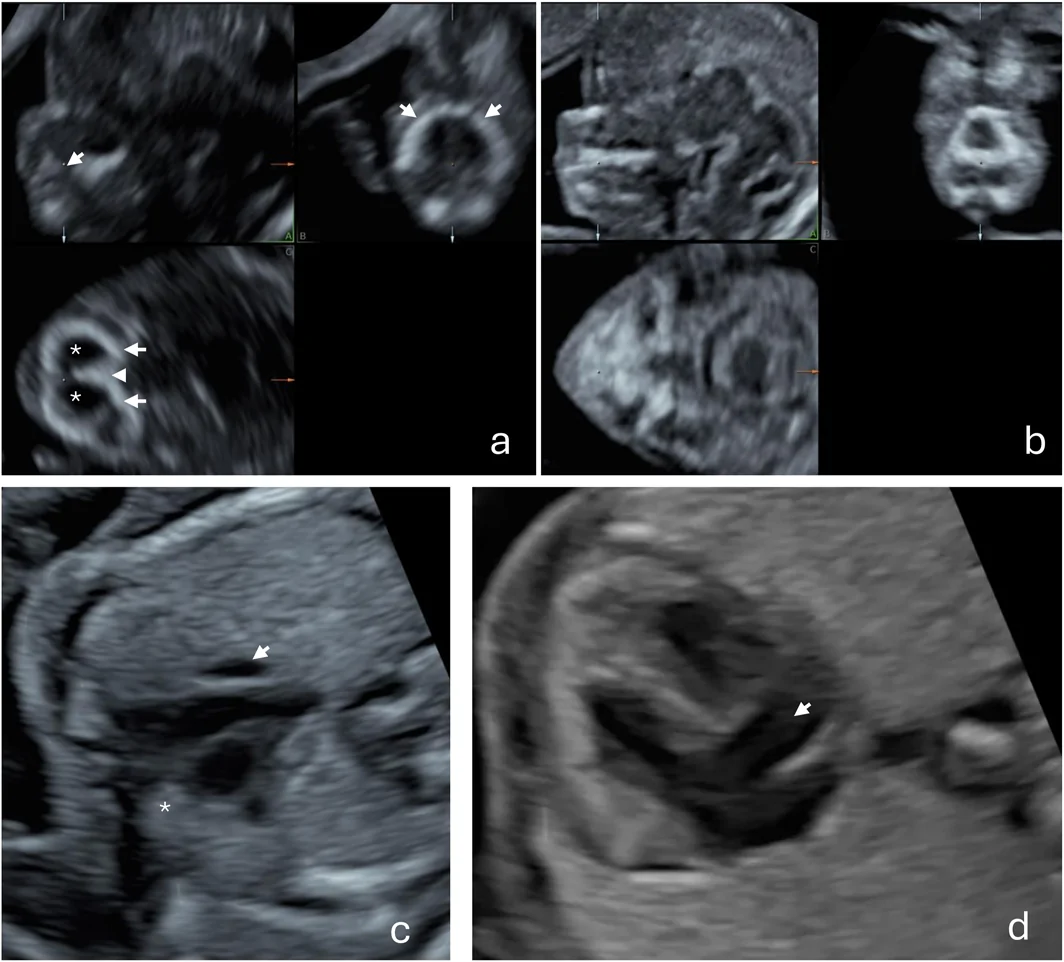
Multiplanar views of the case fetus at 13 weeks of gestation (a) and a gestational‐age–matched unaffected fetus (b) for comparison. A distinct maxillary gap is visible in the sagittal plane (A) (arrow). In the frontal plane (B), there is lateral displacement of the frontal processes of the maxilla, distorting the typical configuration of the retronasal triangle and producing a dome‐like appearance (arrows). The axial plane (C) demonstrates bilateral, symmetric, anechoic dilation of the nasal cavity (asterisks) with well‐defined bony margins, including closed posterior choanae (arrows) and a prominent vomer (arrowhead). Additional axial sections of the fetal thorax at 17 weeks of gestation demonstrate a persistent left superior vena cava (c, arrow), a hypoplastic thymus (c, asterisk), and a dilated coronary sinus (d, arrowhead).

A 42‐year‐old gravida 2, para 1 woman with a naturally conceived dichorionic diamniotic twin pregnancy underwent first‐trimester ultrasound at 13 + 1 weeks. Nuchal translucency measurements and aneuploidy screening were normal in both fetuses. Fetus A had normal morphology, whereas Fetus B showed a maxillary gap, prompting detailed evaluation of the face and head. Three‐dimensional multiplanar imaging demonstrated bilateral symmetric dilatation of the nasal cavities with well‐defined bony margins and a prominent vomer without an associated facial cleft (Figure 1a), raising suspicion of bilateral choanal atresia (*HP:0004502*). Given the twin pregnancy and risk of sampling discordance, follow‐up ultrasound and genetic counseling were scheduled at 17 + 2 weeks. The nasal cavity appearance persisted, and additional findings included a persistent left superior vena cava (*HP:0005301*) draining into a dilated coronary sinus, thymic hypoplasia (thymic‐thoracic ratio 0.27; *HP:0010515*), echogenic bowel (*HP:0010943*), bilateral pyelectasis (*HP:0011129*), and oligohydramnios (Table 1, Figure 1a–d). The parents were nonconsanguineous, with an unremarkable family history. Amniocentesis was performed on the basis of a genetic indication.

**TABLE 1:** A: Clinical data.

Parental details				Gestation at diagnosis	Phenotypes (HPO terms) in fetus B	Obstetric history	Family history	Outcome
Maternal		Paternal						
Age (years)	Ethnicity	Age (years)	Ethnicity					
42	White (European ancestry)	42	White (European ancestry)	13^+1^ weeks	Bilateral choanal atresia (HP:0004502)	Gravida 2, para 1	Unremarkable	Twin A: Spontaneous preterm delivery at 35 weeks; Twin B: Selective fetocide of twin B at 22 weeks gestation. No autopsy due to advanced maceration
				17^+2^ weeks	Bilateral choanal atresia (HP:0004502), Persistent left superior vena cava (HP:0005301), Aplasia/hypoplasia of the thymus hypoplasia (HP:0010515), Echogenic fetal bowel (HP:0010943), Fetal pyelectasis (HP:0010945), Oligohydramnios (HP:0001562),			

## 2 Diagnostic Method

QF‐PCR and chromosomal microarray analysis (CMA) were performed, followed by whole‐exome sequencing (NovaSeq X Plus). Exome analysis was performed using a quad‐based approach including both twins and their parents, with variant prioritization guided by the Human Phenotype Ontology terms listed above.

### 2.1 Diagnostic Results Nad Interpretation

In fetus B, QF‐PCR showed a male fetal sex chromosome pattern with no evidence of common aneuploidies. Chromosomal microarray showed no pathogenic copy‐number variants explaining the phenotype. SNV analysis of WES data identified a *de novo* heterozygous pathogenic missense variant, NM_003482.4: c.10624 C > G p.(Leu3542Val), in exon 38 of the *KMT2D* gene (Figure S1). Pathogenic missense variants in exons 38 and 39 of the *KMT2D* gene have been associated with autosomal dominant Branchial arch abnormalities, choanal atresia, athelia, hearing loss, and hypothyroidism syndrome (BCAHH; OMIM #620186). [1] Table 2 provides a summary of the genetic findings for both fetuses.

**TABLE 2:** Genetic findings.

Procedure (gest age)	Direct/culture?	Performed test	Secondary confirmatory test	Gene (name; REFSEQ)	Known disease (OMIM)	Variant	ACMG classify‐cation	Criteria applied	Inheritance & zygosity	Interpretation
Amniocentesis in fetus B (17 + 2 weeks)	Direct	QF‐PCR	—	—	—	—	—	—	—	No aneuploidy, male sex
	Direct	CMA	—	—	—	—	—		—	No pathogenic CNVs detected
	Direct	SNV analysis of WES data	Sanger sequencing	KMT2D (NM_003482)	Branchial arch abnormalities, choanal atresia, athelia, hearing loss, and hypothyroidism syndrome (OMIM #620186)	c.10624 C > G p.(Leu3542Val)	Pathogenic	PS2, PM1, PM2, PM5, PP2, PP4	Autosomal dominant, heterozygous, de novo	Pathogenic variant consistent with and explanatory of the observed fetal phenotype.
Amniocentesis in fetus A (17 + 2 weeks)	Direct	QF‐PCR	—	—	—	—	—	—	—	No aneuploidy, male sex
	Direct	CMA	—	—	—	—	—		—	No pathogenic CNVs detected
	Direct	SNV analysis of WES data	—	—	—	—	—		—	No clinically relevant variants identified

## 3 Pregnancy Outcome

Follow‐up ultrasound showed severe growth restriction, oligohydramnios, and external ear hypoplasia, in addition to previously identified anomalies. The nasal cavity appeared unremarkable but was difficult to assess. After counseling, the parents elected selective fetocide at 22 weeks of gestation. Twin A was delivered spontaneously at 35 weeks. Advanced maceration after a 13‐week interval precluded autopsy.

## 4 Discussion

Prenatal detection of choanal atresia is rare and has not been reported in the first trimester. Consistent with our findings, Bronstein et al. described transient enlargement of the nasal cavity in the second trimester. [2].

Choanal atresia, a congenital obstruction of the posterior nasal aperture, occurs in approximately 1 in 5000–7000 live births. While one quarter of cases are isolated, bilateral forms are frequently syndromic and most commonly associated with CHARGE syndrome. Rare associations with Kabuki syndrome due to *KMT2D* variants have also been reported. [2, 3] Notably, a patient carrying the same variant as reported here was initially diagnosed with CHARGE syndrome but was postmortem reclassified as having Kabuki syndrome, highlighting the phenotypic overlap between these disorders. [4].

In 2020, Cuvertino et al. described a distinct phenotype associated with pathogenic variants in exons 38–39 of *KMT2D*, characterized by branchial arch anomalies, choanal atresia, athelia, hearing loss, and hypothyroidism [1]. Subsequent reports expanded the phenotype to include developmental delay, growth restriction, and external ear anomalies. [5] The same variant has previously been reported in an infant who presented prenatally with ventricular septal defect, fetal growth restriction, oligohydramnios, echogenic bowel, and small echogenic kidneys. Postnatally, choanal atresia, external and internal ear anomalies, severe combined immunodeficiency, small renal cysts, thyroid abnormalities, and brain malformations were identified; the infant died at 3 months of age. [6].

This case is notable for the first‐trimester identification of an abnormal nasal cavity configuration suggestive of choanal atresia. Because these features may become less apparent with advancing gestation, early imaging offers a narrow diagnostic window. Recognition of this sign enabled the first reported prenatal diagnosis of BCAHH syndrome.

## Funding

This work was supported by the Charles University in Prague (UNCE/24/MED/018).

## Ethics Statement

The authors have nothing to report.

## Consent

The patient consent form was signed by the pregnant woman.

## Conflicts of Interest

The authors declare no conflicts of interest.

## Supporting information


**Figure S1:** Schematic representations of the *KMT2D* c.10624 C > G variant identified in the affected fetus and of the corresponding wild‐type allele in the unaffected family members.

## Data Availability

The data that support the findings of this study are available on request from the corresponding author. The data are not publicly available due to privacy or ethical restrictions.
