# Supplementary material for: First‐Trimester Bilateral Choanal Atresia as a Marker of a De Novo Pathogenic KMT2D Variant Associated With BCAHH Syndrome
Source: Prenat Diagn. 2026 Jul 26;46(10):1667–71. doi: 10.1002/pd.70227 (PMC13571806; doi:10.1002/pd.70227)
Supplement: Supplementary file 1 [file PD-46-1667-s001.pdf]

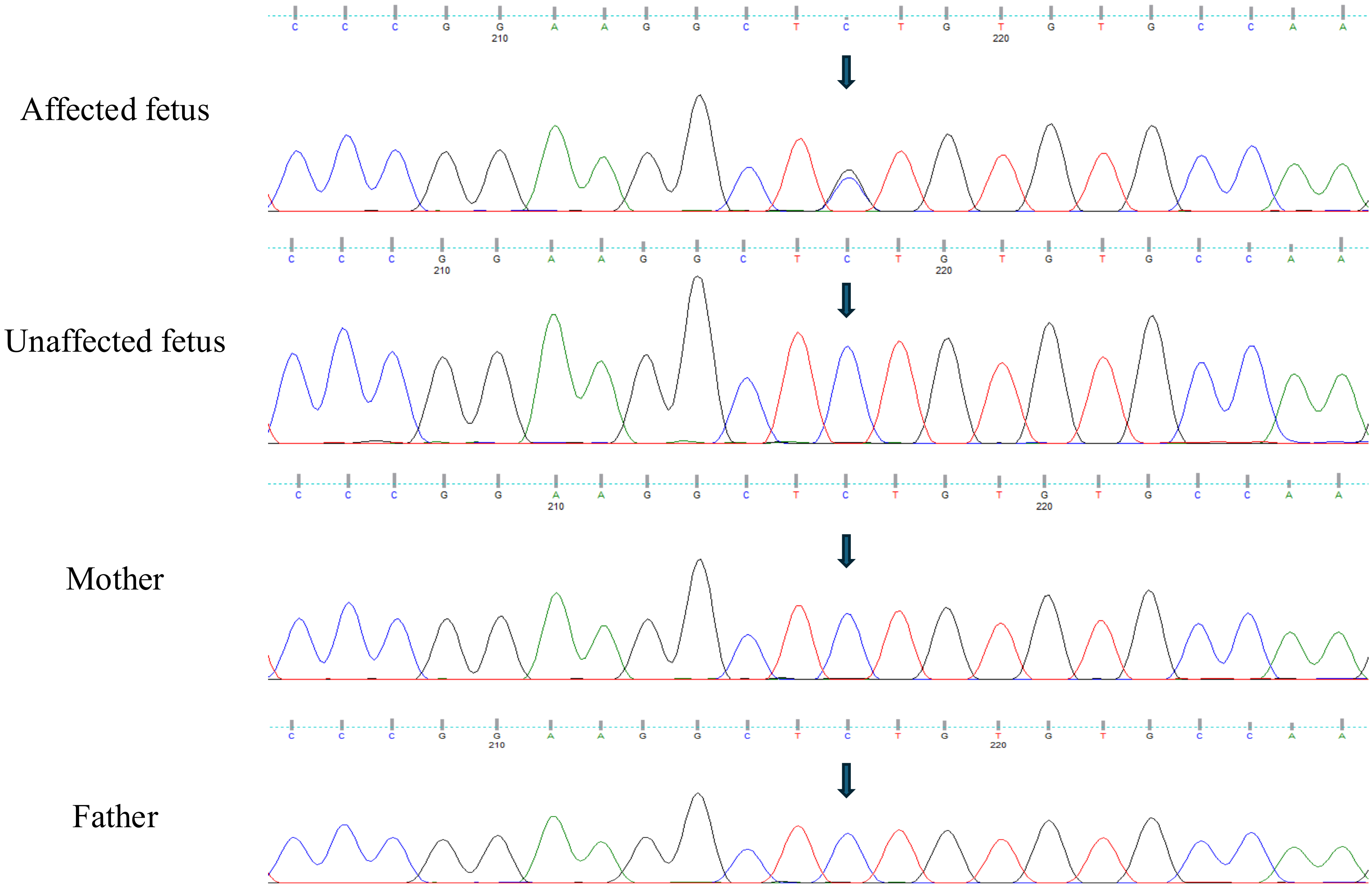
Affected fetus
Unaffected fetus
Mother
Father
